# A Cooperative Interaction between Nontranslated RNA Sequences and NS5A Protein Promotes *In Vivo* Fitness of a Chimeric Hepatitis C/GB Virus B

**DOI:** 10.1371/journal.pone.0004419

**Published:** 2009-02-10

**Authors:** Lucile Warter, Lisette Cohen, Yann Benureau, Deborah Chavez, Yan Yang, Francis Bodola, Stanley M. Lemon, Cinzia Traboni, Robert E. Lanford, Annette Martin

**Affiliations:** 1 Institut Pasteur, Unité de Génétique Moléculaire des Virus à ARN, CNRS URA 3015, Université Paris Diderot - Paris 7 EA 302, Paris, France; 2 Southwest Foundation for Biomedical Research, Southwest National Primate Research Center, San Antonio, Texas, United States of America; 3 Center for Hepatitis Research, Institute for Human Infections and Immunity, University of Texas Medical Branch, Galveston, Texas, United States of America; 4 Istituto di Ricerche di Biologia Molecolare P. Angeletti, Pomezia, Italy; Yonsei University, Republic of Korea

## Abstract

GB virus B (GBV-B) is closely related to hepatitis C virus (HCV), infects small non-human primates, and is thus a valuable surrogate for studying HCV. Despite significant differences, the 5′ nontranslated RNAs (NTRs) of these viruses fold into four similar structured domains (I-IV), with domains II-III-IV comprising the viral internal ribosomal entry site (IRES). We previously reported the *in vivo* rescue of a chimeric GBV-B (vGB/III^HC^) containing HCV sequence in domain III, an essential segment of the IRES. We show here that three mutations identified within the vGB/III^HC^ genome (within the 3′NTR, upstream of the poly(U) tract, and NS5A coding sequence) are necessary and sufficient for production of this chimeric virus following intrahepatic inoculation of synthetic RNA in tamarins, and thus apparently compensate for the presence of HCV sequence in domain III. To assess the mechanism(s) underlying these compensatory mutations, and to determine whether 5′NTR subdomains participating in genome replication do so in a virus-specific fashion, we constructed and evaluated a series of chimeric subgenomic GBV-B replicons in which various 5′NTR subdomains were substituted with their HCV homologs. Domains I and II of the GBV-B 5′NTR could not be replaced with HCV sequence, indicating that they contain essential, virus-specific RNA replication elements. In contrast, domain III could be swapped with minimal loss of genome replication capacity in cell culture. The 3′NTR and NS5A mutations required for rescue of the related chimeric virus *in vivo* had no effect on replication of the subgenomic GBneoD/III^HC^ RNA *in vitro*. The data suggest that *in vivo* fitness of the domain III chimeric virus is dependent on a cooperative interaction between the 5′NTR, 3′NTR and NS5A at a step in the viral life cycle subsequent to genome replication, most likely during particle assembly. Such a mechanism may be common to all hepaciviruses.

## Introduction

Hepatitis C virus (HCV) infection is an important public health concern with about 170 million persons chronically infected worldwide who are at risk for developing liver cirrhosis and hepatocellular carcinoma. To date, the only available treatment is a combination therapy with pegylated interferon-α and ribavirin, which remains poorly effective for HCV genotype 1 infections, and is generally difficult to tolerate, leading to ≥50% therapeutic failures [reviewed in Ref. 1]. The development of specific, small molecule antiviral inhibitors that could be used to cure this infection is thus currently a high priority. Immunocompetent animal models susceptible to HCV infection would be useful to support these efforts and monitor potential antiviral resistance mechanisms. As a surrogate for chimpanzees, which represent the only immunocompetent animal model for human HCV infection but which are endangered and extraordinarily difficult to access, infection of small New World non-human primates from the *Callithrichidae* family (tamarins, marmosets) with a closely related, hepatotropic virus, GB virus B (GBV-B), may prove useful [2], [3]. In most cases, experimental infection of tamarins or marmosets with GBV-B induces an acute hepatitis [2], [4], but in limited cases described to date, experimental GBV-B infection has also resulted in an extended viremia and chronic hepatitis in tamarins [5], [6], mimicking closely chronic hepatitis C in humans.

**Figure 1:**
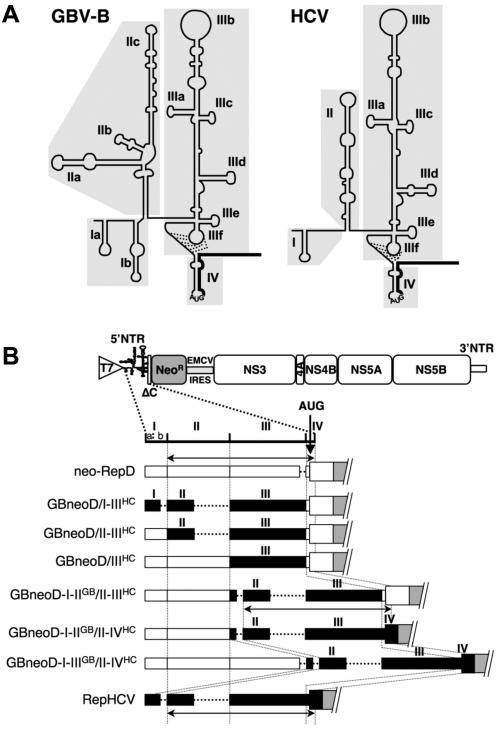
Chimeric GBV-B/HCV replicons engineered. (A) The predicted schematic secondary structures of domains I-IV, each highlighted by shaded areas, in 5′NTRs of GBV-B (left) and HCV (right) are shown. Subdomains forming stem-loops are labeled. Base-pair interactions within an RNA pseudo-knot at the base of domain III are indicated by dotted lines. Core coding sequences downstream of the initiator AUG codon in domain IV are represented by thick lines. (B) The parental GBV-B subgenomic replicon cDNA, neo-RepD [30], cloned downstream of the T7 RNA polymerase promoter is schematically represented at the top of the panel. The first cistron downstream of the GBV-B 5′NTR encodes a fusion polypeptide comprised of the 22 N-terminal core amino acids (ΔC) and neomycine phosphotransferase (Neo^R^), while the second cistron encodes GBV-B nonstructural proteins NS3-NS5B downstream of the murine encephalomyocarditis virus (EMCV) IRES. In the blow up of the 5′NTR, boundaries of domains I, II, III, IV are indicated with the position of the translation AUG initiator codon. For each chimeric 5′NTR, GBV-B and HCV sequences are represented by white and black boxes, respectively, that are drawn to scale according to nucleotide lengths of each domain. Dotted lines indicate shorter domains in one viral 5′NTR with respect to counterparts in the other virus. Arrows below domain schemes delineate IRES sequences.

GBV-B is an enveloped virus that belongs to the *Flaviviridae* family, and has tentatively been classified within the same genus as HCV, the hepacivirus genus [7]. We and others have shown that GBV-B shares a number of features with HCV, but also exhibits some differences, in terms of genomic organization [8], [9], viral protein maturation [10]–[12], and virus/host interactions [13]–[16]. The GBV-B genome contains a single open reading frame encoding a polyprotein which is subsequently cleaved into three structural proteins and seven nonstructural (NS) proteins, among which NS3 to NS5B, only, are required for efficient genome replication in cell culture [17]. The GBV-B and HCV 5′ and 3′ nontranslated RNA (NTR) segments share structural similarities despite significantly divergent nucleotide sequences and lengths. GBV-B and HCV 5′NTRs (445 and 341 nucleotides in length, respectively) are each predicted to fold into four structural domains composed of one or several stem-loops (I, II, III and IV, see Fig. 1A) [18], [19]. Like HCV and pestivirus 5′NTRs, the GBV-B 5′NTR contains an internal ribosomal entry site (IRES) which drives the initiation of cap-independent RNA translation by specific binding of the 40S ribosomal subunit [19], [20]. GBV-B and HCV structural domains II, III and domain IV, extending up to the initiating AUG codon are essential for IRES activity [19], [21], while the first ∼40 nucleotides of the downstream core coding segment may modulate IRES function [22]–[24]. HCV 5′NTR domains I and II have also been shown to both contain essential RNA replication elements, while domain III is not essential, but substantially upregulates HCV RNA replication [25]–[27]. At the other end of the genome, the HCV 3′NTR is composed of a short variable region, followed by a poly(U/UC) tract with 80 nucleotides on average and an almost invariant 98 nucleotide long sequence, the 3′X region. The GBV-B 3′NTR has a similar overall organization, although with a shorter and homopolymeric poly(U) sequence in lieu of the poly(U/UC) tract in HCV, and a larger sequence downstream of the poly(U) stretch comprising 309 nucleotides. The HCV 3′X region, as well as a minimal poly(U/UC) sequence are known to be essential elements required for genome replication [28], [29]. In contrast, the analysis of GBV-B genome replication elements has been hampered by the lack of suitable cell culture models in which this can be studied.

We previously reported that the substitution of domains II-III or I-II-III within the 5′ NTR segment of an infectious molecular clone of GBV-B with the homologous sequences from HCV had a lethal effect on viral replication, preventing the rescue of infectious virus following intrahepatic inoculation of synthetic RNA into tamarins [3]. In contrast, intrahepatic inoculation of a full-length chimeric GBV-B RNA in which only domain III was substituted with its HCV counterpart resulted in the rescue of a chimeric virus (vGB/III^HC^) in tamarins. The genome of the chimeric virus recovered from serum of infected tamarins was found to contain 6 stable nucleotide substitutions, all outside of the chimeric 5′NTR sequence [3]. In the present study, we have analyzed these mutations and show that 3 of the 6 mutations (in the 3′NTR and NS5A coding sequence) are both necessary and sufficient for a robust, wild-type-like viral replication phenotype *in vivo*, and thus presumably compensate for a reduction in replication fitness imposed by the chimeric HCV sequences within domain III of the 5′NTR. To assess the mechanism(s) underlying these compensatory mutations, we have evaluated a series of chimeric RNAs derived from a subgenomic GBV-B replicon which autonomously replicates in human hepatoma cells [30]. We show that domains I and II of the GBV-B 5′NTR contain essential and virus-specific RNA replication signals, whereas domain III, which is essential for virus translation, does not play a critical, virus-specific role in RNA replication. The substitution of domain III in the GBV-B replicon with the corresponding HCV domain resulted in a replication-competent chimeric RNA with only moderately decreased replication capacity in cell culture. We show that the 3′NTR and NS5A substitutions required for robust replication of the corresponding 5′NTR chimeric virus *in vivo* are not required for, nor modulate the RNA replication capacity of the chimeric replicon. These data suggest the existence of a cooperative interaction between the hepacivirus 5′ and 3′ NTRs and NS5A that is essential for a step in the virus life cycle subsequent to genome replication, most likely particle assembly.

## Results

### Mapping RNA replication signals in the GBV-B 5′NTR using chimeric GBV-B/HCV replicons

We previously reported that chimeric, genome-length GBV-B RNAs in which domains I-II-III or II-III of the 5′NTR were substituted by corresponding domains of HCV 5′NTR were not infectious upon intrahepatic inoculation of synthetic RNAs in tamarins [3]. To assess whether this lack of infectivity was due to an RNA replication defect, or a defect in some other step in the virus life cycle, we replaced the different structural domains of GBV-B 5′NTR (Fig. 1A) with the corresponding domains of the H77 strain of genotype 1a HCV [31] within the backbone of a dicistronic, subgenomic GBV-B replicon [neo-RepD, Ref. 30]. In the GBneoD/I-III^HC^ replicon, the nearly entire GBV-B 5′NTR (domains I-II-III) was substituted with HCV domains I-II-III. GBneoD/II-III^HC^ contains sequences comprising most of the HCV IRES (domains II-III) in place of the corresponding GBV-B domains downstream of GBV-B domain I. GBneoD/III^HC^ contains only domain III of HCV in place of the corresponding GBV-B domain downstream of GBV-B domains I-II ; hence, it harbors a chimeric IRES (Fig. 1B). The domain IV sequence comprising 14 nucleotides up to the AUG initiator codon, that modulates IRES activity [24], was derived from GBV-B in all three constructs. These chimeric GBV-B/HCV 5′NTRs thus correspond to those analyzed in the genome-length chimeric RNAs tested previously for viability in intrahepatically-inoculated tamarins [3].

5′NTR sequences may be involved in both RNA replication and translation, and thus the HCV 5′NTR sequences in these chimeric replicons may be required to functionally substitute for GBV-B sequences in providing critical replication signals. However, a defect in RNA replication could theoretically be masked by inefficiencies in translation imposed by the chimeric 5′NTR segments. Therefore, to determine the minimal virus-specific 5′NTR sequences necessary for GBV-B RNA replication, we constructed GBV-B/HCV chimeras in which translation was placed under the control of an intact HCV IRES, while segments of GBV-B 5′NTR potentially involved in genome replication (domains I-II or I-II-III) were retained upstream. These constructs were thus generated with the aim of uncoupling replication from translation signals contained within the GBV-B 5′NTR. The HCV IRES was either nearly complete (domains II-III, with domain IV derived from GBV-B, in GBneoD-I-II^GB^/II-III^HC^), or intact (domains II-III-IV, in GBneoD-I-II^GB^/II-IV^HC^ and GBneoD-I-III^GB^/II-IV^HC^) in these constructs (Fig. 1B). To avoid potential steric hindrance and functional interference between the highly structured GBV-B and HCV domains, a 23-nucleotide-long spacer was inserted upstream of the HCV IRES (see Material and Methods).

**Figure 2:**
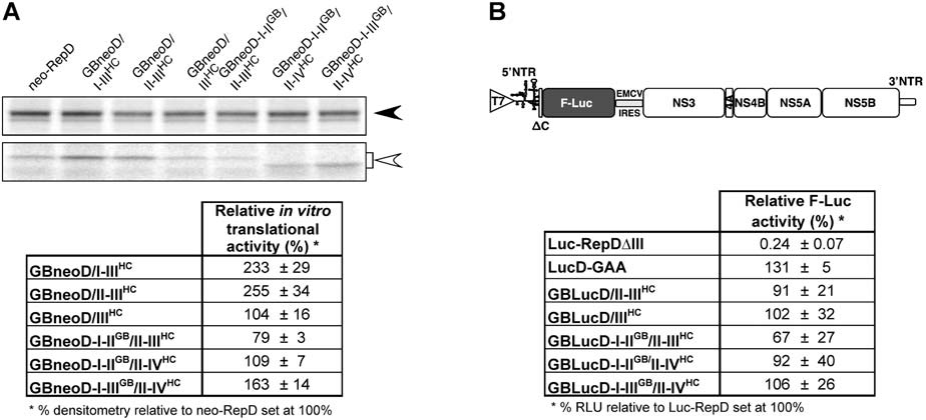
Translational activities of chimeric GBV-B/HCV replicon RNAs. (A) Chimeric replicon RNAs (see Fig. 1) and parental GBV-B replicon RNA neo-RepD were translated *in vitro* in rabbit reticulocyte lysates. A representative PAGE-SDS pattern of the first cistron products, i.e. fusion polypeptides between N-terminal core residues and neomycine phosphotransferase (ΔC-neo, open arrowhead), and of second cistron cleavage products for normalization purposes (GBV-B NS3, filled arrowhead), is shown. Note that ΔC-neo polypeptides have different electrophoretic mobilities depending on the number and nature of N-terminal core residues fused to neo (see text). Translational activities of chimeric replicon RNAs are represented as mean±SD relative PhosphorImager volumes for ΔC-neo products quantified from quadruplicate PAGE-SDS gels and normalized with respect to corresponding NS3 PhosphorImager volumes, expressed relatively to neo-RepD volumes set at 100% (Table). (B) A scheme of the reporter replicons used to monitor translational activities in cells is shown at the top of the panel. The product encoded by the first cistron of this replicon is a fusion protein between N-terminal core residues and firefly luciferase (F-Luc). Control reporter RNAs include a translation-deficient GBV-B F-Luc RNA lacking 5′NTR domain III (Luc-RepDΔIII) and a replication-deficient GBV-B F-Luc RNA with a mutation in the RNA polymerase active site (LucD-GAA). Equal amounts (5 µg) of replicon RNAs were transfected into cB76.1/Huh7 cells. Relative F-Luc activities determined at 4 h post-transfection were expressed after normalization with respect to total protein quantities assayed and relatively to values obtained for parental Luc-RepD, set at 100%. Shown are means±SD of four independent transfections, each monitored in duplicate wells.

The translational activities of the chimeric GBV-B/HCV replicon 5′NTRs were first monitored in rabbit reticulocyte lysates, in the presence of microsomal membranes such as to optimize the processing of the viral nonstructural polyprotein NS3-NS5B synthesized from the second cistron of the replicons [32]. GBV-B/HCV IRESs present in the chimeric replicons drive the synthesis of either of two fusion polypeptides consisting of 22 GBV-B core codons (when domain IV is derived from GBV-B) or 12 HCV core codons (when domain IV is derived from HCV) fused to neomycin phosphotransferase (ΔC-neo) (see Fig. 1B). A typical electrophoresis pattern of *in vitro* translated NS3 and ΔC-neo polypeptides, as well as quantitation of ΔC-neo products normalized with respect to the synthesis of NS3 (produced from the second cistron under translational control of the EMCV IRES) in replicate experiments are shown in Fig. 2A. Normalization with respect to another cleavage product from the second cistron (NS4B) gave similar values (not shown). These data demonstrated that the *in vitro* translational efficiencies of the different chimeric 5′NTRs with domain swaps (GBneoD/I-III^HC^, GBneoD/II-III^HC^, GBneoD/III^HC^) were equivalent to or higher than that of the parental neo-RepD. The translational activities of these chimeric replicon RNAs were thus in good agreement with data previously obtained for similar chimeric 5′NTRs driving the synthesis of a reporter protein (renilla luciferase) encoded by monocistronic transcripts [3]. Replicons in which replication and translation activities were uncoupled (GBneoD-I-II^GB^/II-III^HC^, GBneoD-I-II^GB^/II-IV^HC^, GBneoD-I-III^GB^/II-IV^HC^) also demonstrated translational competence, although with decreased efficiency compared to chimeras in which domains II-III were swapped, in particular for GBneoD-I-II^GB^/II-III^HC^ (Fig. 2A). This could possibly be due to partial (GBneoD-I-II^GB^/II-III^HC^, GBneoD-I-II^GB^/II-IV^HC^) or complete (GBneoD-I-III^GB^/II-IV^HC^) redundancy of GBV-B and HCV IRES elements that may compete for the binding of translational factors.

Since cellular factors may modulate chimeric GBV-B/HCV RNA translation, we next assayed translational efficiencies of the various chimeric 5′NTRs in cell culture. For that purpose, GBV-B permissive cells, derived from the Huh7 human hepatoma cell line [cB76.1/Huh7 cells, Ref. 17] were transfected with chimeric replicons encoding firefly luciferase (F-Luc) as a reporter protein in place of neomycin phosphotransferase (Fig. 2B). Quantification of F-Luc at 4 hours post-transfection, normalized with respect to total protein quantities, reflected translational activities of input RNAs prior to the onset of RNA replication. Relative luciferase activities were in the same range for all five chimeric replicons tested, as well as the parental GBV-B F-Luc replicon and a replication-incompetent GBV-B F-Luc RNA mutant harboring mutations in the sequence encoding the RNA polymerase active site (LucD-GAA) (Fig. 2B). As expected, a control GBV-B replicon RNA lacking 5′NTR domain III (Luc-RepDΔIII) produced background levels of luciferase activity (Fig. 2B). It is worth noting that the translational activity of GBneoD-I-II^GB^/II-III^HC^ was slightly reduced (67–79%), as compared to the parental neo-RepD and chimeric GBneoD-I-II^GB^/II-IV^HC^ levels, both *in vitro* (Fig. 2A) and in cells (Fig. 2B), highlighting the contribution of homologous domain IV to HCV IRES activity. Altogether, these results show that the various chimeric 5′NTRs retain translational competence in Huh7 cells with no marked differences in efficiency.

**Figure 3:**
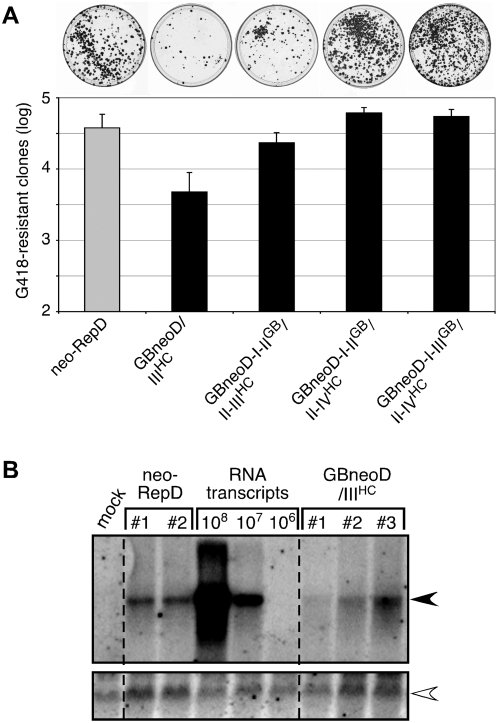
Replication activities of chimeric GBV-B/HCV replicon RNAs. (A) G418-resistant colony forming activities of the indicated chimeric replicons (black bars, see Fig. 1) are expressed as means±SD of log values obtained in ≥4 independent transfections (2×10^6^ cells transfected with 5 µg RNA) performed with ≥3 independent RNA transcript syntheses, relatively to that of parental GBV-B replicon (grey bar) set at the mean value±SD obtained throughout transfection experiments. Typical patterns of G418-resistant cell clones stained at 3 weeks post-transfection from 100-mm dishes in which 3×10^4^ transfected cells were plated are shown above the graph. (B) Total RNA was extracted from 2–3 cell clones individually picked and expanded after transfection with chimeric replicon GBneoD/III^HC^ (see Fig. 1) or parental GBV-B replicon neo-RepD and analyzed by Northern blot with a riboprobe specific for GBV-B positive-strand RNA after electrophoresis on a denaturing agarose gel. As controls, RNA from mock-transfected cells (mock), as well as various quantities of neo-RepD synthetic RNA transcripts (10^6^, 10^7^, 10^8^ genome equivalents) mixed with cellular RNA from mock-transfected cells, were loaded on the same gel and processed in parallel. Viral RNA and housekeeping β-actin mRNA, detected by a specific riboprobe as a loading control, are indicated by filled and open arrowheads, respectively. Dotted lines indicate where noncontiguous lanes that belong to the same Northern blot image have been brought together.

To analyze their replication capacity, the chimeric GBV-B/HCV RNA transcripts were transfected into cB76.1/Huh7 cells and cells were maintained under G418 selective pressure for 3 weeks. No replicon containing G418-resistant cell clone was observed after transfection of the GBneoD/I-III^HC^ or GBneoD/II-III^HC^ RNA (data not shown). These results thus show that the substitution of domains I-II-III or II-III of GBV-B by corresponding HCV domains within the backbone of a GBV-B subgenomic replicon abrogates RNA replication in cell culture, while RNA translation was unaffected (Fig. 2). These results also indicate that the lack of infectivity of genome-length RNAs with similar chimeric 5′NTRs following intrahepatic inoculation in tamarins, as reported previously [3], was due to a major defect in RNA replication. In sharp contrast, numerous G418-resistant cell clones were obtained following transfection of the GBneoD/III^HC^ RNA, although approximately 7-fold less, compared to the parental GBV-B replicon neo-RepD (Fig. 3A). This moderately reduced replication capacity cannot be attributed to a translational defect of the chimeric 5′NTR (Fig. 2). Eight cell clones were isolated and expanded from GBneoD/III^HC^ transfections and viral RNAs present in these clones were analyzed by real-time quantitative RT-PCR. GBneoD/III^HC^ RNA was detected in all clones with an abundance (6.11±0.17 log genome equivalents/µg total RNA) similar to that found in cell clones containing the parental neo-RepD RNA (5.88±0.03). The integrity of GBneoD/III^HC^ RNAs present in cell clones was verified by Northern blot detection of viral RNA after electrophoresis on denaturing gels (Fig 3B). Viral RNA extracted from 5 cell clones was reverse-transcribed, PCR-amplified and sequenced in its entirety, with the exception of the 40 5′ and 100 3′ terminal nucleotides, respectively. In all cases, the chimeric nature of the 5′NTR was confirmed and no mutation was found from the initial chimeric replicon sequence (data not shown). These data indicate that the substitution of domain III resulted in a replication-competent chimeric replicon, although with moderately decreased (∼7-fold lower) colony forming efficiency compared to neo-RepD. This supports the infectivity of a genome-length GBV-B chimera harboring a similar domain III substitution, as observed upon intrahepatic inoculation of synthetic RNA [3].

To further investigate whether only domains I-II of GBV-B contain critical, virus-specific RNA replication elements, we similarly analyzed the chimeric replicons in which translation was uncoupled from replication and no longer dependent on the GBV-B 5′NTR. As shown in Fig. 3A, the number of G418-resistant cell clones obtained after transfection of cB76.1/Huh7 cells with GBneoD-I-II^GB^/II-III^HC^ or GBneoD-I-II^GB^/II-IV^HC^ replicon was in the same range as that obtained with neo-RepD. Colony formation was ∼2.5-fold less efficient with GBneoD-I-II^GB^/II-III^HC^ than with GBneoD-I-II^GB^/II-IV^HC^ (Fig. 3A), which might be correlated with decreased translational activity (Fig. 2B). Previous studies of HCV replicons in which translation was placed under the control of poliovirus IRES, with various lengths of 5′-terminal HCV sequences were retained upstream, indicated that the 5′NTR domain III in HCV contains important replication signals that substantially enhance RNA replication [25], [26]. To determine whether sequences within GBV-B domain III could also modulate RNA replication, we assessed the colony forming efficiency of GBneoD-I-III^GB^/II-IV^HC^, which differs from GBneoD-I-II^GB^/II-IV^HC^ by the addition of GBV-B domain III upstream of the HCV IRES. As shown in Fig. 3A, the number of cell clones obtained with GBneoD-I-III^GB^/II-IV^HC^ replicon did not significantly differ from that obtained with GBneoD-I-II^GB^/II-IV^HC^. Sequences of chimeric replicon RNAs recovered from pools of G418-resistant cell clones at 3 weeks post-transfection were shown to correspond to input sequences in noncoding regions, indicating that partially duplicated 5′NTRs were not rearranged upon RNA replication. Taken together, these results suggest that domains I and II of the GBV-B 5′NTR, but not domain III, contain essential, virus-specific RNA replication elements.

### Analysis of second site mutations found in the genome of a domain III chimeric virus rescued *in vivo*: compensatory effect on chimeric genome infectivity *in vivo*

In previous studies, we found that replacing domain III in an infectious molecular clone of GBV-B with the corresponding HCV domain (GB/III^HC^) resulted in an infectious genome with rescue of a chimeric virus following intrahepatic inoculation of RNAs into a susceptible tamarin, yet with an unusual delay in the onset of viremia. The rescued virus (vGB/III^HC^) was subsequently shown to give rise to rapid and robust viremia upon passage into a naïve animal, and to contain six stable nucleotide substitutions when compared to the input chimeric RNA [3]. Thus, given the lengthy delay in the onset of viremia in the first tamarin following intrahepatic inoculation of RNA, it is likely that these mutations compensate for an incompatibility between the HCV domain III sequence and the GBV-B sequence comprising the balance of chimeric genome. These mutations include two substitutions leading to amino acid changes in the p13 and NS5A coding sequences (U_2376_C: Ile_644_Thr; and U_7152_C: Val_2236_Ala, respectively), two substitutions in the 3′NTR segment located upstream of the poly(U) sequence (C_9052_U, and C_9065_U), and two silent changes in the E1 and NS5B coding sequences (G_1012_A, A_7945_G, respectively) (numbering refers to nucleotide position within genome-length GBV-B RNA, see Table 1) [3].

**Table 1:** Summary of the infectivity of mutated chimeric GB/III^HC^ genomes *in vivo* and sequencing of corresponding viruses

Tamarins	E1^a^	p13 a	NS5A a	NS5B a	3′NTR a	Infectivity^b^	Virus mutations^c^
T16453	G_1012_A	U_2376_C	U_7152_C	A_7945_G	C_9052_U/C_9065_U	+	ΔC_238_, G_5225_A
T16452	—	U_2376_C	U_7152_C	—	C_9052_U/C_9065_U	+	G_5225_A
T16448	—	—	U_7152_C	—	—	−	NA
T16468	—	—	U_7152_C	—	—	−	NA
T16457	—	—	—	—	C_9052_U/C_9065_U	+/−	NA
T16463	—	—	—	—	C_9052_U/C_9065_U	+/−	NA
T16459	—	—	U_7152_C	—	C_9052_U/C_9065_U	+	—
T16440	—	—	U_7152_C	—	C_9052_U/C_9065_U	+	ΔC_238_, C_5226_U

a Combinations of nucleotide substitutions introduced into chimeric genome GB/III^HC^ in the indicated genomic regions (—: no substitution). Numbering refers to nucleotide position within genome-length GBV-B RNA. Substitutions U_2376_C and U_7152_C lead to amino acid changes Ile_644_Thr and Val_2236_Ala, respectively.

b The infectivity of mutated chimeric RNAs was assessed by intrahepatic inoculation of corresponding synthetic RNAs into 1 or 2 tamarins and was scored “+” when viremia developed according to wild-type-like profile and peak viremia was ≥3.5×10^7^ ge/mL, “+/−” when viremia was sporadic and ≤2.5×10^4^ ge/mL, and “−” when viremia remained negative or ≤1.5×10^3^ ge/mL at 1–2 scattered time points.

c Mutations additionally found in the corresponding chimeric viruses rescued *in vivo* (numbering in the 5′NTR refers to nucleotide position within the chimeric GBV-B/HCV 5′NTR). Substitutions G_5225_A and C_5226_U lead to amino acid changes Ala_1594_Thr and Ala_1594_Val, respectively. —: no mutation was found in the sequenced regions. NA: not applicable, either because there was no virus rescued or because the viral titers were too low to proceed to sequencing.

**Figure 4:**
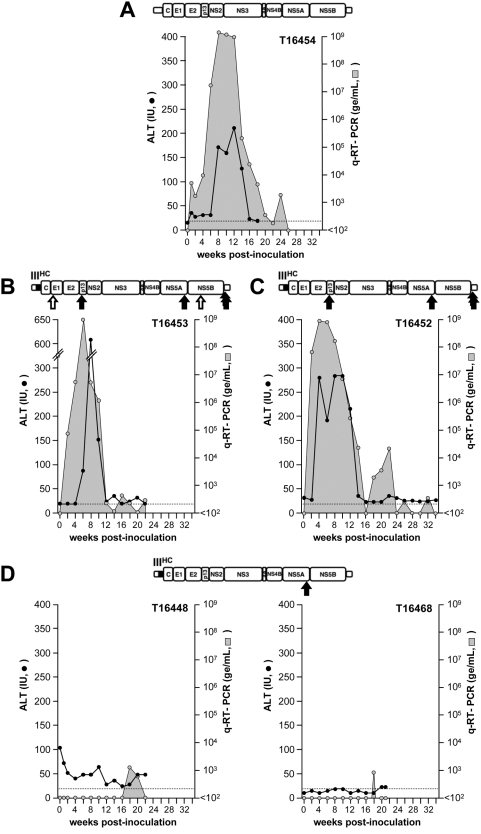

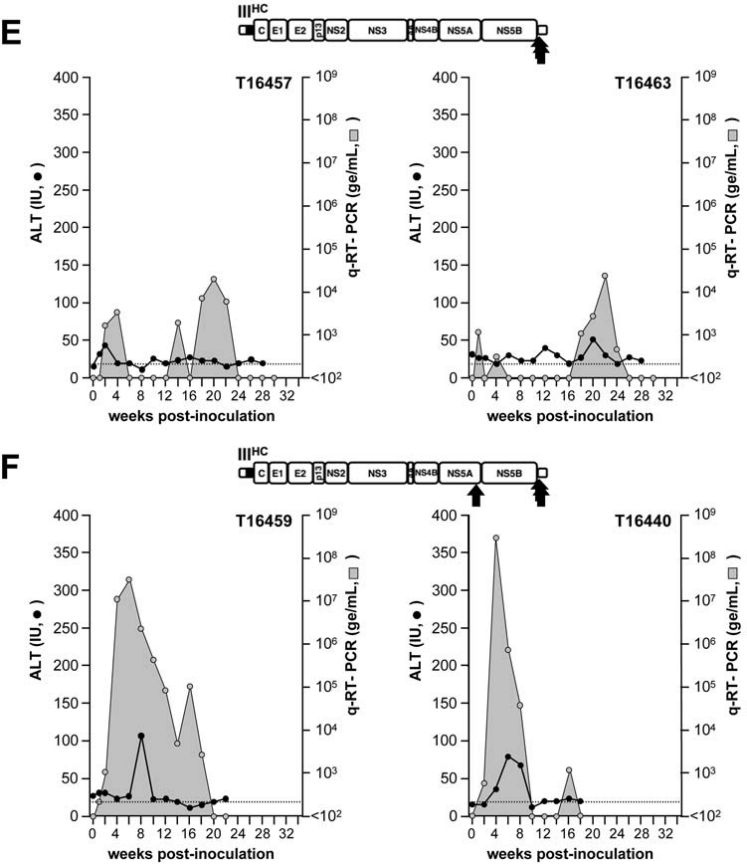
*In vivo* infectivity of mutated chimeric RNAs. Synthetic, genome-length RNA transcripts derived from (A) the parental GBV-B infectious molecular clone [5] or (B–F) 5′NTR domain III chimeras containing all or combinations of the 6 nucleotide substitution(s) found in the chimeric virus rescued *in vivo*, were inoculated individually into the liver of GBV-B-naïve tamarins (B, C, and right graphs in panels D–F) or tamarins that previously received nonreplicating GBV-B-derived RNAs (A and left graphs in panels D–F). Nucleotide substitutions (see Table 1) are depicted by open arrows (silent changes) or filled arrows (coding changes or changes in the 3′NTR) under the schematic genome inserted above each graph (B–F). The extent of viremia (shaded area) was monitored over time in the serum of each animal by quantitative (q)-RT-PCR (genome equivalents, (ge)/mL), as well as by analysis of alanine aminotransferase (ALT) activity (black dots, international units, IU). ALT baseline levels are indicated on each graph by a dotted line.

To determine which of these mutations were able to compensate for the putative defect in the chimeric GBV-B/HCV genome, we introduced either one or a combination of these nucleotide substitutions into the chimeric GB/III^HC^ genome and assessed the infectivity of each corresponding synthetic RNA by intrahepatic inoculation into a GBV-B-naïve tamarin (Fig. 4). In some cases, we also tested infectivity in a second tamarin, using animals that had previously received a nonreplicating GBV-B-derived construct to minimize the number of naïve animals used in these studies. We first demonstrated that the inclusion of all 6 nucleotide changes was able to confer an immediate, robust *in vivo* replication phenotype to the chimeric RNA (tamarin T16453, Fig. 4B), similar to the pattern observed after inoculation of the parental, wild-type GBV-B RNA (T16454, Fig. 4A). A combination of the two non-silent nucleotide substitutions in p13 and NS5A, respectively, together with the two 3′NTR substitutions also conferred a robust replication phenotype to the chimeric genome (T16452, Fig. 4C). This strongly suggests that the two silent changes in E1 and NS5B coding sequences are not required for a high level of replication fitness of the chimeric virus.

In other studies, we have found that the NS5A change was consistently selected for in all viruses rescued *in vivo* from the wild-type GBV-B molecular clone used here [3], [5], therefore we hypothesized that this mutation was not directly compensating for a sequence incompatibility within the chimeric genome. Indeed, the inclusion of this single substitution did not enhance the infectivity of the GB/III^HC^ chimeric genome in two animals tested (T16448 and T16468, Fig. 4D). Unlike the NS5A mutation, the two 3′NTR mutations have never been observed in viruses rescued *in vivo* from the wild-type molecular clone, suggesting they may be in fact true compensatory mutations. When introduced together in the absence of the other mutations, the 3′NTR mutations were not fully compensatory, but they did confer a low level of replication, with sporadic detection of viremia (≤2.5×10^4^ ge/mL) at multiple time points, (T16457 and T16463, Fig. 4E). However, there was no further adaptation of the virus before the animals cleared the virus. The titer of virus in these animal sera were not sufficient to allow viral genome sequencing. Finally, inclusion of the two 3′NTR mutations, together with the NS5A substitution, fully compensated for the replication defect introduced by the chimeric 5′NTR and resulted in a parental-type virus replication profile in two tamarins (compare T16440 and T16459, Fig. 4F, with T16454, Fig. 4A). We conclude from these studies that the combination of the 3′NTR and NS5A substitutions is both necessary and sufficient for robust replication of the chimeric virus, but that (as described above) the NS5A substitution is likely to confer a general increase in replication fitness independent of the presence or absence of HCV sequence within the 5′NTR (Table 1).

The genomes of viruses recovered at different time points from tamarins T16440, T16459, T16452, T16453 were sequenced in regions of interest (5′NTR; p13, NS4A and NS5A coding sequences; 3′NTR). The chimeric nature of the 5′NTR and the presence of engineered substitutions were confirmed in all cases. Nucleotide U_2376_ in the p13 coding sequence that was found mutated towards a C in the genome of vGB/III^HC^ was not mutated in the genomes of T16440 and T16459 viruses at any time point (not shown). This confirms that the U_2376_C substitution encoding an Ile_644_Thr amino acid change in p13 is not required for adaptation of the chimera to efficient replication *in vivo*. The mutated NS5A coding sequence did not evolve further in any virus (not shown). Interestingly, we observed a deletion of 1 C (nt 238) among a stretch of 7 cytosine residues at the 5′ end of HCV domain III in virus recovered from T16440 at early time points (week 4 post-inoculation (p.i.)), and in virus from T16453 at later time points (week 8 p.i.) (Table 1). A potentially beneficial effect of this mutation on virus fitness is suggested by the fact that it was also identified in the original vGB/III^HC^ recovered at late time points from the RNA-inoculated animal (data not shown), although it was not selected for in other chimeric viruses that replicated efficiently *in vivo* (T16452 and T16459 viruses) (Table 1). No mutations were identified in the 3′NTR sequence upstream of the poly(U) tract in chimeric viruses, except in T16453 virus recovered at late time points (≥week 8 p.i), in which there was a mixture of A (wild-type-like) and G (mutated) residues at nucleotide 9035 (not shown). This mutation has not been found in the genome of wild-type GBV-B recovered from 5 RNA-inoculated tamarins, and thus its significance is unknown. Interestingly, a C_5226_U nucleotide substitution encoding an Ala_1594_Val mutation in NS4A was identified in T16440 virus, while a G_5225_A substitution encoding an Ala_1594_Thr mutation at the same position was observed in T16452 and T16453 viruses recovered at late time points. No change in NS4A coding sequence was observed in T16459 virus (Table 1). Similar changes at this NS4A residue have been observed in viruses recovered from animals inoculated with wild-type GBV-B RNA transcripts (viruses from 2 tamarins had an Ala/Val change, viruses from 2 other animals had an Ala/Thr change, and virus from 1 animal had no change, data not shown). Thus these mutations, like the U_7152_C substitution in NS5A, are likely to reflect a suboptimal sequence of the GBV-B molecular clone at this position, and unlikely to be a specific compensatory response to the inclusion of HCV sequence within the chimeric 5′NTR. Taken together, the data thus indicate that the C_9052_U and C_9065_U substitutions in the 3′NTR are clearly compensatory and specifically related to the presence of HCV sequence in the 5′NTR, while no other nucleotide change was subsequently found to confer increased fitness specifically to the 5′NTR chimeric virus.

### Effect of *in vivo* compensatory mutations on 5′NTR chimeric RNA translation and replication

**Figure 5:**
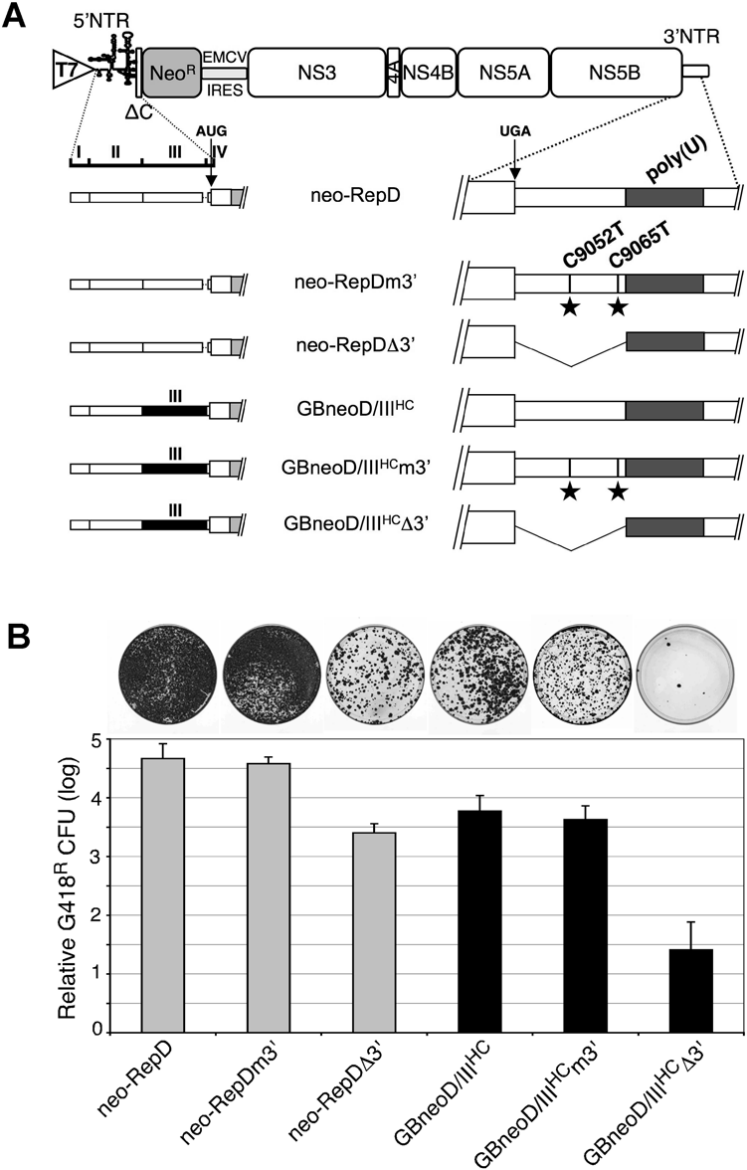
Replication activities of replicons with mutated 3′NTR. (A) The chimeric or mutated nature of the 5′ and 3′ NTRs of the indicated chimeras is depicted on the left and right of the figure, respectively, below the GBV-B subgenomic replicon scheme (see legend to Fig. 1). In the 5′NTRs, white and black boxes correspond to GBV-B and HCV sequences, respectively. Within the 3′NTR of GBV-B, dark grey boxes correspond to the poly(U) tract, stars to the indicated nucleotide substitutions (numbering refers to nucleotide positions within GBV-B genome-length cDNA), and broken lines to the extent of the nucleotide deletion. Positions of translation initiator (AUG) and termination (UGA) codons are indicated by arrows. (B) G418-resistant colony forming activities of the indicated RNAs with a GBV-B 5′NTR (grey bars) or a chimeric 5′NTR containing HCV domain III (black bars) are expressed as means±SD of log values obtained in ≥4 independent transfections (2×10^6^ cells transfected with 5 µg RNA) performed with ≥3 independent RNA transcript syntheses, relatively to that of neo-RepD set at the mean value±SD obtained throughout transfection experiments. Typical patterns of G418-resistant cell clones stained at 3 weeks post-transfection from 100-mm dishes in which 5×10^5^ transfected cells were plated are shown above the graph for each replicon.

To explore the mechanism(s) underlying the compensatory effect of these mutations on the *in vivo* replication fitness of the chimeric virus, we investigated whether these mutations enhance the translation and/or replication fitness of the corresponding chimeric RNA replicons. We first focused on the two substitutions in the 3′NTR, which were introduced into the cDNA of the chimeric replicon GBneoD/III^HC^, as well as that of the parental GBV-B replicon neo-RepD, as a control (giving rise to GBneoD/III^HC^m3′ and neo-RepDm3′, respectively, Fig. 5A). In addition, in order to evaluate the potential contribution of the 3′NTR region located upstream of the poly(U) sequence to genome replication, we introduced a deletion of 27 nucleotides, extending from the polyprotein stop codon to the poly(U) sequence, into both GBneoD/III^HC^ and neo-RepD cDNAs (giving rise to GBneoD/III^HC^Δ3′ and neo-RepDΔ3′, respectively, Fig. 5A). Similar F-Luc replicons were constructed and transfected into cB76.1/Huh7 cells, in order to determine whether these 3′NTR mutations or the 3′NTR deletion modulated viral RNA translation in cell culture. As shown in Table 2, luciferase activities measured 4 hours after transfection were similar for all these replicons, suggesting that there is likely no contribution of the 3′NTR domain upstream of the poly(U) sequence to GBV-B RNA translation.

**Table 2:** Translational activities of 3′NTR mutant replicons in cB76.1/Huh7 cells

Replicon^a^	Relative F-Luc activity (%) b
Luc-RepDm3′	107±41
Luc-RepDΔ3′	129±47
GBLucD/III^HC^	102±32
GBLucD/III^HC^m3′	175±20
GBLucD/III^HC^Δ3′	127±66
Luc-RepDΔIII	0.24±0.07
LucD-GAA	131±5

a Replicon RNAs containing 3′NTR mutations or deletions correspond to those depicted in Fig. 5A, but encode firefly luciferase (F-Luc) in place of neomycine phosphotransferase. Luc-RepDΔIII and LucD-GAA control RNAs are described in the legend of Fig. 2.

b F-Luc RLU activities were determined 4 hours after transfection of equal amounts (5 µg) of replicon RNAs into cB76.1/Huh7 cells, and expressed relatively to Luc-RepD set at 100% after normalization with respect to total protein quantities assayed (mean±SD from two independent transfections, each monitored in duplicate wells).

Next, replication activities of these 3′NTR mutated replicons were compared by monitoring colony forming activities in transfected cB76.1/Huh7 cells. The deletion of the 27 3′NTR nucleotides between the polyprotein stop codon and poly(U) sequence strongly affected GBV-B replication, with ∼20-fold and ∼150-fold reductions in colony formation for neo-RepDΔ3′ and GBneoD/III^HC^Δ3′ replicons, respectively, compared to their parental neo-RepD and chimeric GBneoD/III^HC^ replicon counterparts (Fig. 5B). These data suggest that the 3′NTR segment upstream of the poly(U) stretch contributes substantially to genome replication, but is not essential. The deletion of this 3′NTR segment appeared more deletorious in the context of the domain III chimeric replicon than in the parental GBV-B backbone, but the significance of this is uncertain. Importantly, and in strong contrast to what we found with the full-length RNA constructs in tamarins, the two 3′NTR mutations had no effect on the replication competence of either the parental or chimeric subgenomic GBV-B replicon RNAs (compare neo-RepDm3′ and neo-RepD, as well as GBneoD/III^HC^m3′ and GBneoD/III^HC^, Fig. 5B). We sequenced GBneoD/III^HC^m3′ RNA present in a pool of cell clones harvested at 3 weeks post-transfection, and demonstrated that these RNAs retained the chimeric 5′NTR and mutated 3′NTR, with no other consistent change in the NTRs. Thus, these studies revealed that the two mutations at nucleotides 9052 and 9065 in the 3′NTR segment upstream of the poly(U) sequence do not modulate the replication efficiency of the chimeric GB/III^HC^ RNA in cell culture.

The U_7152_C mutation resulting in a Val_2236_Ala change in NS5A that we identified in the vGB/III^HC^ genome is naturally present in the neo-RepD and GBneoD/III^HC^ replicon cDNAs, as these were constructed from different infectious molecular clones. Indeed, the molecular clone from which neo-RepD is derived differs from the molecular clone used in our *in vivo* studies by 38 nucleotides scattered throughout the genome, with the exception of the 5′NTR and 3′NTR segment upstream of the poly(U) sequence. We introduced a C_7152_U mutation in neo-RepD to match the sequence of our GBV-B infectious clone at this position, and found that this substitution had no effect on replicon colony forming efficiency (data not shown). This result argues that this substitution within the NS5A coding sequence is likely not to act at the level of genome replication.

Altogether, these data suggest that the cooperative effect of the NS5A and 3′NTR mutations on the replication fitness of the 5′NTR chimeric virus is likely to act at a step in the virus life cycle that is subsequent to viral RNA translation and genome replication.

## Discussion

GBV-B is the only other tentative member of the hepacivirus genus, along with HCV, and a model virus that can be used *in vivo* in small primates to mimic certain aspects of HCV infection. In this study, a GBV-B subgenomic replicon allowed the identification of nontranslated RNA sequences involved in GBV-B genome replication. This replicon (neo-RepD) [30] is derived from an infectious molecular clone that induces a robust viremia upon inoculation of synthetic RNAs in the liver of tamarins [33]. While GBV-B infects small New World primates like tamarins, marmosets and owl monkeys, but not chimpanzees [34], hence probably not humans either, neo-RepD replicon RNA is adapted to replicate in human hepatoma, Huh7-derived cells [30].

We found no significant differences in the translational activities of replicon RNAs containing chimeric HCV/GBV-B sequences engineered within the backbone of GBV-B subgenomic replicon (Fig. 1), either in rabbit reticulocyte lysates (Fig. 2A) or in cell culture (Fig. 2B), regardless of whether translation was driven from a chimeric GBV-B/HCV IRES (II^GB^/III^HC^/IV^GB^ or II-III^HC^/IV^GB^), or intact HCV or GBV-B (II-III-IV) IRESs. Also, we observed no differences in translational activity when domains II-III of both GBV-B and HCV were fused to each other with an intervening spacer sequence (GBneoD-I-III^GB^/II-IV^HC^), despite the potential for competition for essential translational factors. However, it should be noted that there was a minor but significant enhancing effect on translation when domain IV was derived from the same virus as the sequence in domains II-III of the IRES (GBneoD-I-II^GB^/II-IV^HC^ versus GBneoD-I-II^GB^/II-III^HC^, Fig. 2). Conflicting data have been reported in the literature with respect to the putative role of the 3′NTR on the regulation of HCV RNA translation, ranging from no effect [28], [35], [36], to a stimulatory effect [37] or inhibitory effect [38]. A recent study suggested that these conflicting findings arose from the use of different viral RNA systems, including reporter mini-genomes, dicistronic subgenomic replicon RNAs, or full-length genomes [39]. Song *et al.* concluded that, in the case of HCV, the three structural regions of the 3′NTR including the variable region, the poly(U/C) tract and the 3′-X region all contribute significantly to HCV IRES activity, suggesting that a functional interaction between 5′ and 3′ NTRs is involved in HCV RNA translation [39]. Our findings for GBV-B show that the 3′NTR segment upstream of the poly(U) tract does not modulate GBV-B IRES activity in subgenomic parental or chimeric GBV-B/HCV replicons (Table 2).

Structural predictions suggest that domain I of the GBV-B 5′NTR folds into two stem-loops, whereas domain I of HCV 5′NTR is shorter and is predicted to fold into a single 5′ terminal stem-loop [18], [19]. A mutational study of an HCV subgenomic replicon suggested that the 5′-proximal stem-loop of the HCV RNA is an essential *cis*-acting RNA element for HCV genome replication [40]. We show here that domain I of GBV-B 5′NTR is also essential and provides virus-specific RNA replication elements that cannot be substituted by the corresponding HCV proximal stem-loop I. The present study also shows that domain II of the GBV-B 5′NTR is critical for RNA replication, in addition to its known role in translation. The involvement of both domains I and II in genome replication is shared by HCV, a feature that was previously reported using HCV subgenomic replicons in which translation of the first cistron was placed under the control of poliovirus IRES [25], [26]. Reusken et al. reached the same conclusion by showing that subgenomic HCV RNAs failed to replicate when domains II or II-III were replaced by the corresponding domain(s) from a pestivirus 5′NTR [27].

Domains III of GBV-B and HCV exhibit only ∼60% nucleotide identity, but do share similar lengths and structural folds [19] and were shown here to be exchangeable with minimal loss of GBV-B replicon colony formation (Fig. 3). In addition, we did not observe enhancement in colony formation with a chimeric replicon in which GBV-B domains I through III were present upstream of the HCV IRES (GBneoD-I-III^GB^/II-IV^HC^), as compared to the chimeric replicon in which only domains I-II of GBV-B were present upstream of the HCV IRES (GBneoD-I-II^GB^/II-IV^HC^) (Fig. 3), while all of these replicons had similar translational activities (Fig. 2). In contrast, the addition of domain III to domains I-II of HCV 5′NTR upstream of the poliovirus IRES in HCV subgenomic replicons increases by 100-fold HCV replicon colony formation in Huh7 cells [25], [26]. Thus, while both GBV-B and HCV contain replication signals that overlap translation signals in domain II, 5′NTR sequences involved in RNA replication of GBV-B do not appear to extend into domain III, in contrast to that of HCV. Our data suggest, however, that GBV-B domain III might be involved in regulating a switch between translation and replication when both activities are coupled, since a chimeric II^GB^-III^HC^ segment ensuring both translation and replication (in GBneoD/III^HC^) provides parental-type translational activity, but not full replication capacity. Alternatively, we cannot rule out the possibility that the presence of the HCV domain III might modify the folding of the GBV-B domain II in this chimeric construct, thereby potentially affecting important GBV-B replication signals. However, M-FOLD-assisted structural modeling of the GBneoD/III^HC^ 5′NTR did not suggest that there are changes in the folding of domain II in this chimeric 5′NTR (not shown). Despite these differences between GBV-B and HCV domain III requirement for genome replication, 5′NTR sequences involved in genome replication of these hepaciviruses in cell culture both differ strikingly from those in pestiviruses, for which only the most 5′ four nucleotides appear essential for RNA replication, and only domain I is required for efficient replication [41]–[43].

We also investigated the role of the 3′NTR segment upstream of the poly(U) stretch in GBV-B genome replication. In HCV, the deletion of the proximal 23–24 nucleotides of the variable domain upstream of the poly(U/C) segment in the 3′NTR did not hamper genome infectivity *in vivo*, in chimpanzees [44], but impaired subgenomic RNA replication [29]. Interestingly, a genome-length GBV-B RNA bearing a 27 nucleotide-deletion from stop codon to poly(U) sequence was readily infectious in tamarins [6], while we show here that the GBV-B subgenomic replicon bearing a similar deletion (neo-RepDΔ3′) exhibits a significantly reduced (∼20-fold) colony formation activity (Fig. 5B). Although GBV-B genome-length RNAs and subgenomic replicons were not derived from identical genetic backgrounds, it is unlikely that a ∼7-fold decrease in colony formation activity, as observed here for subgenomic domain III chimeric RNA GBneoD/III^HC^, could account for the lengthy delay in replication of the corresponding genome-length chimeric RNA *in vivo* in tamarins [3]. In line with this, while we showed that 3 nucleotide substitutions in NS5A coding sequence and 3′NTR were necessary and sufficient for adaptation of the genome-length domain III chimera to efficient replication in tamarins (Fig. 4, Table 1), we did not find these mutations to enhance genome translation or replication in the replicon model (Table 2, Fig. 5B). Altogether, assuming that Huh7-derived cells and tamarin hepatocytes provide similar cellular factors involved in GBV-B genome replication, our data suggest that these adaptive mutations may act to facilitate a later step in the virus life cycle, possibly involving packaging and/or particle assembly.

The involvement of both 3′NTR sequences and NS5A protein in hepacivirus assembly is not unprecedented. Studies using the JFH1 strain of HCV in cell culture [45], [46] indicate that the C-terminal domain III of NS5A is essential for assembly of infectious particles. The mechanisms of action of NS5A in particle assembly are not fully understood yet, but they appear to involve the ability of NS5A to co-localize together with the core protein on lipid droplets, a cellular organelle that was recently discovered to play a key role in HCV particle assembly [47]. Although the structure and function of GBV-B NS5A have not yet been investigated, it is interesting to note that the Val_2236_Ala substitution that we found in vGB/III^HC^ maps to the C-terminus of GBV-B NS5A, in a region that aligns with domain III of HCV NS5A. Packaging signals are not known for HCV or GBV-B, but could well involve core protein/RNA interactions. A segment of the conserved 3′X domain of the HCV 3′NTR has been suggested to be involved in RNA dimerization in an interaction that is chaperoned by the core protein [48], [49]. The authors of these studies found that the 3′X segment was able to adopt several alternative monomeric or dimeric conformations, and proposed that interconversions between these various forms might constitute a molecular switch that could regulate translation/replication/packaging of the positive-strand viral RNA. Similarly, the GBV-B core protein appears to have a chaperone activity that facilitates stable RNA structures and 3′NTR dimerization, although potential 3′NTR domains involved in these structures have not been described [50]. It is tempting to speculate that the 3′NTR mutations that we have shown here provide fitness to the chimeric vGB/III^HC^ virus in tamarins do so by facilitating a cooperative interaction with the chimeric 5′NTR and possibly with the C-terminal region of NS5A. Such an interaction could either regulate a molecular switch shifting viral RNA from replication to packaging, or otherwise facilitate infectious GBV-B particle assembly. An immortalized primate hepatocyte cell line susceptible to GBV-B infection would help address this potential mechanism, which may be common to all hepaciviruses.

## Materials and Methods

### Cells

Human hepatoma cells cB76.1/Huh7 [17], derived from HuH-7 cells [51], were cultured in Dulbecco's modified Eagle's medium supplemented with 100 U of penicillin per mL, 100 µg of streptomycin per mL, and 10% fetal calf serum (DMEM-10%).

### Plasmids

The GBV-B neo-RepD plasmid [30] contains a dicistronic subgenomic GBV-B cDNA which encodes a fusion polypeptide comprised of the 22 N-terminal amino acids of core fused to neomycin phosphotransferase (*neo*) (which confers resistance to G418) under the translational control of GBV-B IRES, and GBV-B NS3-NS5B under the translational control of the EMCV IRES (Fig. 1B). The replicon cDNA is inserted downstream of a unique *Bam*HI restriction site followed by the T7 RNA polymerase promoter, and upstream of a unique *Sap*I restriction site that allows the production of an exact 3′ terminus of the viral cDNA for transcription purposes. A unique *Asc*I restriction site is placed between GBV-B core codons and the *neo* gene. A GDD-to-GAA-mutated neo-RepB cDNA [neo-GAA, Ref. 17] was used as a source of nonreplicative RNA in the experiments.

Chimeric segments from previously described plasmids pGB/I-II-III^HC^, pGB/II-III^HC^, and pGB/III^HC^ that contain genome-length GBV-B cDNA with chimeric GBV-B/HCV 5′NTRs [3] were PCR-amplified using a sense primer hybridizing upstream of the T7 RNA polymerase promoter and an anti-sense primer hybridizing to the appropriate GBV-B core codons followed by an engineered 3′-terminal *Asc*I restriction site. PCR fragments were then digested with *Bam*HI and *Asc*I and substituted to the corresponding fragment in neo-RepD, yielding plasmids pGBneoD/I-II-III^HC^, pGBneoD/II-III^HC^, and pGBneoD/III^HC^. To construct plasmid pGBneoD-I-II^GB^/II-III^HC^, overlapping PCR was used on neo-RepD or HCV 1a H77 cDNA [31] templates to fuse GBV-B 5′NTR domains I-II (nucleotides 1–236) to nucleotides 21–43 from HCV 5′NTR domain I (which fold as a non-based-paired RNA sequence, playing the role of a spacer sequence, see Fig. 1A), followed by HCV 5′NTR domains II-III (nucleotides 44–330) and GBV-B domain IV and first 22 core codons (nucleotides 435–511). The PCR fragment was then digested with *Bam*HI and *Asc*I and introduced into the neo-RepD plasmid in place of the analogous segment. An overlapping PCR fragment containing GBV-B domains I-II (nucleotides 1–236) fused to HCV domains II-III-IV and first 12 core codons (nucleotides 21–377) was generated with a 3′ extension containing an engineered *Asc*I restriction site, then digested by *BamH*I and *Asc*I and substituted to the corresponding *BamH*I-*Asc*I fragment into plasmid neo-RepD, yielding pGBneoD-I-II^GB^/II-IV^HC^. Similarly, a *BamH*I/*Asc*I-restricted PCR fragment containing GBV-B domains I-II-III (nucleotides 1–434) fused to HCV domains II-III-IV and first 12 core codons (nucleotides 21–377) was introduced into plasmid neo-RepD, generating pGBneoD-I-III^GB^/II-IV^HC^ (see Fig. 1).

Primer-based mutagenesis and overlapping PCR were used to introduce substitutions C9052T and C9065T (numbering refers to nucleotide position within GBV-B genome-length cDNA) into the 3′NTR of neo-RepD and GBneoD/III^HC^ cDNAs. The resulting PCR fragment was digested with *Bgl*II (GBV-B nucleotide 8629) and *Xho*I (downstream of the 3′ *Sap*I site) and cloned within these restriction sites into neo-RepD and pGBneoD/III^HC^ in place of the corresponding fragments to generate pneo-RepDm3′ and pGBneoD/III^HC^m3′, respectively. An overlapping PCR was performed to delete nucleotides 9041–9067 within the GBV-B 3′NTR, downstream of the termination codon and upstream of the poly(U) stretch. The deleted *Bgl*II-*Xho*I restriction fragment was then introduced into neo-RepD and pGBneoD/III^HC^ plasmids in place of the analogous segments to generate pneo-RepDΔ3′ and pGBneoD/III^HC^Δ3′, respectively.

To construct parental and chimeric reporter replicon cDNAs encoding firefly luciferase (F-Luc) in place of *neo*, the F-Luc gene was PCR-amplified with primers harboring *Asc*I and *Pme*I 5′ and 3′ extensions, respectively, and cloned between *Asc*I and *Pme*I (located immediately upstream of the EMCV IRES) restriction sites of the corresponding *neo* replicon cDNAs.

Primer-based mutagenesis and overlapping PCR were used to introduce point substitutions in genome-length, chimeric 5′NTR cDNA GB/III^HC^ [3] (see Fig. 4 and Table 1). Appropriate unique restriction sites surrounding each targeted nucleotide residue were used to introduce the mutated PCR fragments in lieu of the corresponding parental restriction fragments.

### *In vitro* transcription

Subgenomic replicon and genome-length cDNAs were linearized with *Sap*I and *Xho*I, respectively, prior to *in vitro* transcription using *MEGAscript T7 Kit* (Ambion) according to the manufacturer's instructions. For subgenomic replicons only, the DNA template was removed by treatment with *TURBO DNase* (Ambion) and RNAs were purified by phenol-chloroform extractions and precipitated with ethanol. RNA quality and yields were estimated by electrophoresis on nondenaturing agarose gels with respect to known quantities of an RNA molecular weight marker (*RNA millenium Markers formamide*, Ambion). RNAs were stored at −80°C.

### *In vitro* translation reactions

*In vitro* transcribed RNAs (200 ng) were used to program translation reactions in *Flexi™* rabbit reticulocyte lysates (Promega) in the presence of 6 µCi of [^35^S]-methionine (GE Healthcare), 125mM KCl and canine pancreatic microsomal membranes (Promega). Ten-microliter reactions were incubated at 30°C for 4 h. One-tenth of each reaction was mixed with *LDS sample buffer* and analyzed by electrophoresis on *NuPAGE 4–12% Bis-Tris gels* in *MOPS* buffer (Invitrogen). Gels were then fixed and dried, and translation products were quantitated by PhosphorImager (*STORM 820*, Molecular Dynamics).

### Transfection of cultured cells and extraction of viral RNA

cB76.1/Huh7 cells (2×10^6^ cells) were transfected by electroporation (EasyjecT Plus, EquiBio) with 5 µg of *in vitro* transcribed RNA, as previously described [52]. Quality and quantity of the transfected RNAs were double-checked on agarose gels on the transfection day. Transfected cells were seeded in 100 mm-diameter dishes at variable densities and, when applicable, supplemented with cells transfected with the replication-deficient RNA neo-GAA to reach a final density of 5×10^5^ cells per dish. Twenty-four hours after electroporation, medium was replaced by DMEM-10% containing 0.25 mg/ml of G418 (Invitrogen) and changed thereafter twice a week. At 3 weeks post-transfection, G418-resistant cells clones supporting viral RNA replication were either stained with a 0.1% cristal violet solution or selected and expanded under selective pressure. Total RNA was extracted from cell clones grown to confluence in 100-mm dishes using *TRIzol* reagent (Invitrogen) following the manufacturer's instructions. RNA pellets were dissolved in 30 µl of RNase-free water and RNA yields were quantified by optical density measurements.

### Quantitative RT-PCR analysis of viral RNA

Viral RNA present in G418-resistant cells were quantitated by real-time, 5′-exonuclease RT-PCR (*TaqMan*) using 50 ng of total RNA and a one-step procedure based on the *one-Step RT-PCR Master Mix Reagent* kit (Applied Biosystems) and primers and probe specific for GBV-B NS5A coding sequence, as previously described [5]. RT was performed at 48°C for 30 min, followed by an incubation at 95°C for 10 min and 40 cycles each comprised of 15 s at 95°C and 1 min at 60°C. An *in vitro* transcribed subgenomic GBV-B RNA, quantitated by optical density measurement, was used as a standard. Products were analyzed on an *ABI PRISM™7700 Sequence Detector* (Applied Biosystems).

### Northern blot analysis

Total RNAs (7.5 µg) isolated from G418-resistant cells clones were separated by electrophoresis on denaturating agarose-formaldehyde gels and transferred overnight by capillarity to nylon membranes (*Hybond-N+*, GE Healthcare) in SSC 10× buffer. RNAs were immobilized on membranes by UV-cross-linking (700 µJ/cm^2^). Membranes were then prehybridized for 1 h at 65°C in SSC 5× buffer containing 50% formamide, 0.5% SDS and Denhardt 5×, then hybridized overnight at 65°C in the same buffer supplemented with an α-[^32^P]-labeled antisense riboprobe specific for the GBV-B NS5B coding sequence. A β-actin-specific antisense riboprobe was used to normalize total RNA amounts loaded on the gel. Membranes were then washed 3 times at room temperature in SSC 2×, SDS 0.1%, then 3 times at 74°C in SSC 0.1×, SDS 0.1%. Specific RNAs were visualized by PhosphorImager (*STORM 820*, Molecular Dynamics).

### Luciferase assays

cB76.1/Huh7 cells (2×10^6^ cells) were transfected as described above with 5 µg of *in vitro* transcribed F-Luc replicon RNAs. Fractions of the transfected cells (8×10^4^ cells) were seeded in duplicates in 24-well plates. Four hours after transfection, cells were washed with PBS and lyzed with 125 µl of *Reporter Lysis Buffer* (Promega). Ten µl of cleared lysates were mixed with 50 µl of *Luciferase Assay Reagent* (Promega) and luciferase activity was immediatly measured for 10 s in a luminometer (Lumat LB 9507, Berthold Technologies). For normalization purposes, total protein contents of the lysates were determined by using the *Micro BCA™ Protein Assay Kit* (Pierce).

### Infectivity assay in tamarins; Ethics statement

Tamarins (*Saguinus mystax*) were housed at the Southwest National Primate Center at the Southwest Foundation for Biomedical Research (San Antonio, Texas) and cared for in accordance with the *Guide for the Care and Use of Laboratory Animals*. Experimental protocols were approved by the Institutional Animal Care and Use Committee of the Southwest Foundation for Biomedical Research. The Southwest Foundation has full accreditation from Association for Assessment and Accreditation of Laboratory Animal Care International and conducts research in compliance with national and international standards. Individual animals each received an intrahepatic inoculation of RNA transcripts from a single genome-length construct, to assess their infectivity. The liver was exposed by a limited abdominal incision and approximately 100 µg of RNA inoculated directly into the liver. The quality of the RNA dilution inoculated intrahepatically was subseqeuntly re-checked on a non-denaturing agarose gel on the day of the inoculation. At 7–14 day intervals p.i., serum was collected and assayed for alanine aminotransferase (ALT) activity and viral RNA levels. For the latter, RNA was isolated using a QiaAmp viral RNA extraction kit (Qiagen) and quantitated by real-time, 5′ exonuclease RT-PCR (TaqMan) using a one-step procedure with the TaqMan EZ core reagent kit (Applied Bisosystem) and primers located within the GBV-B NS5A sequence, as described above.

### Sequence analysis of viral RNAs

Viral RNAs extracted from G418-resistant cell clones or from tamarin sera were reverse transcribed using an anti-sense primer specific for nucleotides 9363–9379 in the GBV-B 3′NTR or random hexamer oligonucleotides and *Superscript II* reverse transcriptase (Invitrogen). Purified cDNAs were PCR-amplified using *Platinum Taq* DNA polymerase (Invitrogen) or *pfu* DNA polymerase (Stratagene) and several primer pairs designed to span the GBV-B subgenomic replicon sequence and generate ∼1000–2000 bp-long PCR fragments. Reactions were set up for 35 cycles at 94°C for 30 s, 55°C for 30 s, 70°C for 1 min. Sequencing reactions were programmed with uncloned PCR fragments using *Big Dye terminator version 1.1* kit (Applied Biosystems) and analyzed on ABI 3700 capillary or 373XL DNA sequencers (Applied Biosystems).
